# Electrospun Silk-ICG Composite Fibers and the Application toward Hemorrhage Control

**DOI:** 10.3390/jfb15090272

**Published:** 2024-09-19

**Authors:** Ayesha Siddiqua, Elwin Clutter, Olga Garklavs, Hemalatha Kanniyappan, Rong R. Wang

**Affiliations:** 1 Department of Chemistry, Illinois Institute of Technology, Chicago, IL 60616, USAhkanniyappan@iit.edu (H.K.); 2 Wilbur Wright College, City Colleges of Chicago, Chicago, IL 60634, USA; opgarklavs@gmail.com

**Keywords:** silk fibroin, indocyanine green, electrospinning, near-infrared, hemorrhage control, photothermal effect

## Abstract

In trauma and surgery, efficient hemorrhage control is crucial to avert fatal blood loss and increase the likelihood of survival. There is a significant demand for novel biomaterials capable of promptly and effectively managing bleeding. This study aimed to develop flexible biocomposite fibrous scaffolds with an electrospinning technique using silk fibroin (SF) and indocyanine green (ICG). The FDA-approved ICG dye has unique photothermal properties. The water permeability, degradability, and biocompatibility of Bombyx mori cocoon-derived SF make it promising for biomedical applications. While as-spun SF-ICG fibers were dissolvable in water, ethanol vapor treatment (EVT) effectively induced secondary structural changes to promote β-sheet formation. This resulted in significantly improved aqueous stability and mechanical strength of the fibers, thereby increasing their fluid uptake capability. The enhanced SF-ICG interaction effectively prevented ICG leaching from the composite fibers, enabling them to generate heat under NIR irradiation due to ICG’s photothermal properties. Our results showed that an SF-ICG 0.4% fibrous matrix can uptake 473% water. When water was replaced by bovine blood, a 25 s NIR irradiation induced complete blood coagulation. However, pure silk did not have the same effect. Additionally, NIR irradiation of the SF-ICG fibers successfully stopped the flow of blood in an in vitro model that mimicked a damaged blood vessel. This novel breakthrough offers a biotextile platform poised to enhance patient outcomes across various medical scenarios, representing a significant milestone in functional biomaterials.

## 1. Introduction

Hemorrhage, also known as uncontrolled bleeding, can arise as a result of severe trauma or surgical procedures and is a primary contributor to substantial blood loss and impaired wound healing. This presents a significant threat in emergency, hospital, and battlefield environments, leading to elevated mortality rates [1,2]. Hemostasis is the fundamental phase of wound healing that plays a critical role in halting bleeding via clot formation in injured blood vessels [3]. Several topical hemostats, sealants, and adhesives have been created to achieve hemostasis in surgical settings [4,5,6]. Fibrin glues are the sole FDA-approved substance utilized in operating rooms [7]. Nevertheless, fibrin-based treatments may be washed away by intense blood flow, and excessive use might result in infections or hinder wound healing [8,9]. Alternatively, cyanoacrylates are utilized. However, they may trigger allergic reactions. Their fast-curing properties, arising from their high exothermic reactivity with natural materials, such as cotton, wool, leather, and skin, even from direct contact, can lead to rapid, excessive amounts of heat generation to cause serious burns [10,11]. Despite its limitations, suturing remains the preferred form of treatment for wound closure due to its ability to lower the risk of dehiscence and its overall fast bleeding control when compared to tissue adhesives [12]. Hence, it is imperative to create a therapeutic strategy that can effectively manage hemorrhage to minimize blood loss [13,14].

Generating silk composites to enhance functionality is a subject of particular interest to our research group. Silk fibroin (SF), derived from the cocoons of Bombyx mori silkworms, is a versatile material that has drawn substantial attention in biomedical uses [15,16,17]. This natural protein possesses biocompatibility, biodegradability, and exceptional mechanical qualities, rendering it suitable for various medical applications [17,18,19,20]. SF scaffolds in tissue engineering assist cell proliferation and tissue regeneration [21,22]. Porosity facilitates the exchange of nutrients and oxygen, which enhances cell survival and tissue development [23,24]. SF’s robust mechanical strength and adaptability make it appropriate for constructing intricate tissues such as skin, bone, and cartilage [25,26,27]. Its capacity to encapsulate and release medications in a regulated fashion is beneficial for targeted treatments [15,25,28,29,30]. Interestingly, Kai et al. demonstrated that the aggregation-induced fluorescence of sulfonic groups in artificial spider silk offers a novel way to generate luminescent and robust fibers for surgical and diagnostic biomedical applications [27]. Inspired by spider-silk threads, Liu et al. developed a biomimicking sensing suture (BSS) produced from naturally derived silk proteins. While maintaining clinically significant mechanical properties and biocompatibility, the BSS accomplishes strain sensing, controlled drug delivery, and antibacterial activity [20]. SF drug delivery systems can safeguard delicate pharmaceuticals from degradation, enhance their bioavailability, and improve treatment effectiveness while minimizing negative effects [31,32,33]. SF’s biocompatibility and biodegradability make it ideal for use in implants, including sutures, wound dressings, and tissue scaffolds [34,35,36]. Its mechanical characteristics can be adjusted to align with those of particular tissues, hence decreasing the possibility of implant rejection or dysfunction [37].

Indocyanine green (ICG) is an FDA-approved tricarbocyanine dye commonly used in medical diagnosis due to its beneficial absorption at 780 nm and emission at 820 nm in the near-infrared (NIR) region [38,39,40]. These characteristics allow for deep signal penetration and reduce interference with tissue autofluorescence [41,42,43,44]. ICG also efficiently absorbs NIR light and transforms it into thermal energy using a non-radiative photothermal relaxation process [45,46,47]. However, ICG’s application potential is hindered by drawbacks, including inadequate solubility in aqueous solutions, concentration-dependent aggregation, fast degradation, short circulation half-life, and poor photostability [48,49,50]. Utilizing the benefits of SF and ICG, multiple groups explored the combination of ICG with SF nanoparticles (SF-NPs) to overcome the aforementioned limitations [51,52,53]. For instance, ICG encapsulation in SF-NPs was found to effectively stabilize ICG for photothermal treatment (PTT) and delayed ICG release under NIR light, as opposed to using free ICG solutions [52]. Using supercritical fluid technology, Chen et al. generated SF nanoparticles containing indocyanine green (ICG-SFNPs) with improved ICG photothermal stability and pH-sensitive release of ICG in the acidic tumor environment. The ICG-SFNPs efficiently eradicated cancer cells in laboratory settings and living organisms via light-induced hyperthermia [54].

In this work, SF-ICG composite fibers were generated by electrospinning—a straightforward, inexpensive, and fast method for producing microfibers with the desired composition and structure. This technique expands upon our prior work, in which we adjusted the parameters and spinning conditions to create functional biocomposite fibers for targeted applications. For instance, we generated aligned collagen–silk composite fibers with balanced biochemical and biophysical characteristics to induce rapid neural differentiation of stem cells [55,56]. We also employed electrospinning to incorporate a minute amount of carbon nanotube (CNT) into spider silk protein and silk fibroin, capitalizing on the increased electrical conductivity of the biocomposite fibers to aid in the activation of patients’ dysfunctional fibroblasts by electrical stimulation and boosting collagen synthesis for connective tissue regeneration [57,58]. 

**Figure 1:**
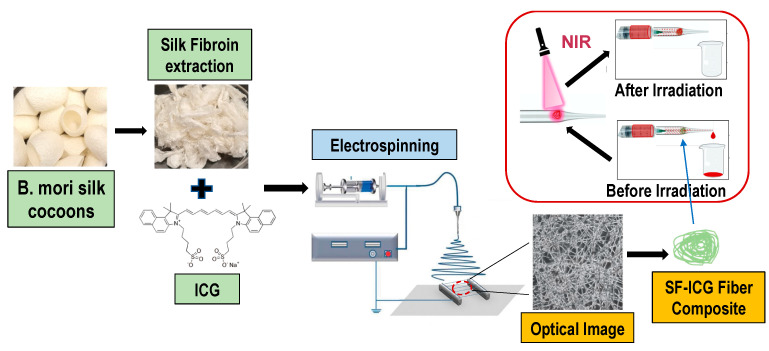
Schematic illustration showing the preparation of SF-ICG electrospun fibers and their application toward hemorrhage control.

Herein, we applied the electrospinning technique and produced networked fibrous matrices of SF-ICG with a high surface-area-to-volume ratio, porosity, flexibility, and tunable composition. In response to a 25 s NIR irradiation, an SF-ICG (0.4%) fibrous matrix effectively harnessed thermal energy release and induced complete blood coagulation to stop blood flow (Figure 1). The developed material and method offer a unique biotextile platform for effective hemorrhage control and the advancement of functional biomaterials for diverse medical applications.

## 2. Materials and Methods

### 2.1. Regeneration of Silk Fibroin

*Bombyx mori* silkworm cocoons were obtained from Aurora Silk, Portland, OR. The cocoons were degummed with 0.02 M Na_2_CO_3_ solution for 1 h at 100 °C, then rinsed with distilled water thrice. Degummed silk was dissolved in an aqueous solution of 9.3 M LiBr at 60 °C for 3 h and then dialyzed at room temperature in a regenerated cellulose tubular membrane (MWCO 6–8 kDa, Fisher Scientific, Pittsburgh, PA, USA) against distilled water for 3 days. The dialyzed SF solution was centrifuged, and the supernatant was frozen at −80 °C overnight and then lyophilized using a Freeze Mobile 6 Lyophilizer (The Virtis Company Inc., Gardiner, NY, USA) to obtain the dry powder [58].

### 2.2. Solution Viscosity Measurement

The viscosity of the 100 mg/mL pure SF and SF-ICG (0.1%, 0.2%, 0.3%, 0.4%, 0.5%, and 0.6%) solutions prepared in 1,1,1,3,3,3-hexafluoro-2-propanol (HFIP) (Oakwood Chemical, Estill, SC, USA) was measured using a DV3T Rheometer (Ametrk Brookfield, Middleborough, MA, USA) at 25 °C. A CP-40 spindle with a speed of 5–13 rpm was used. Before each solution viscosity measurement, the instrument was auto-zeroed and calibrated using deionized (DI) water. A 0.5 mL solution was added to the spindle cup for individual measurement, and the samples were measured in triplicates. 

### 2.3. Preparation of Electrospun SF-ICG Fibers 

A 100 mg/mL SF solution was prepared by dissolving lyophilized SF in HFIP. A 4 mg/mL stock solution of ICG was prepared by dissolving ICG powder in HFIP. A minute amount of ICG solution was added to the SF solution to prepare the SF-ICG solutions containing 0.1–0.6% weight percent of ICG in SF, designated as SF-ICG 0.1–0.6%. A home-built electrospinning setup was used to fabricate the fibers, as described in our previous work [56]. A syringe pump (Harvard Bioscience Inc., Holliston, MA, USA) powered by a high-voltage power supply (Glassman High Voltage, High Bridge, NJ, USA) was used to pump 50 μL pure SF or SF-ICG solution from an 18 G needle (Global Medical Products, Port St Lucie, FL, USA) at a flow rate of 4 mL/h and a voltage of 25 kV. Dense, woven fibers were collected on the surface of a grounded aluminum electrode placed 6 inches below the needle tip. 

The as-spun fibers were water-soluble. To improve their stability in aqueous solution, the fibers were vapor-treated using 70% ethanol (*v*/*v*). First, the as-spun fibers of pure SF or SF-ICG were dried overnight in a vacuum at −20 mmHg to remove the HFIP traces. Subsequently, they were subjected to overnight ethanol vapor treatment (EVT) followed by overnight drying in a vacuum chamber at −20 mmHg. 

### 2.4. Optical Imaging and Fiber Diameter Characterization

The phase and fluorescent images of the as-spun and EVT fibers were collected using a Leica DMi8 Microscope (Leica Microsystem CMS GmbH, Wetzlar, Germany) equipped with a Cy7 excitation filter (Chroma Technology Corp, Bellows Falls, VT, USA) and a Leica DFC9000sCMOS camera. The excitation wavelength range was 672–748 nm, and the emission wavelength was in the range of 765–855 nm. Fiber diameters were measured using the ImageJ (1.53k/Java 1.8.0_172 (64-bit)) and OriginPro (2021b (64-bit) SR1 9.8.5.204) software. Diameters of 120 fibers were measured for each sample to achieve statistical analysis. 

### 2.5. FTIR Spectroscopy

Attenuated total reflection Fourier transform infrared (ATR-FTIR) spectra of electrospun (E-spun) fibers were collected using a Thermo Nicolet Nexus 470 FTIR spectrometer (Madison, WI, USA) equipped with a single-bounce diamond ATR crystal accessory. The spectra were collected at a nominal resolution of 4 cm^−1^. Peak deconvolution of the amide I region was carried out following the procedure developed by Kreplak et al. [59] to evaluate the protein fibers’ secondary structural changes. In brief, the secondary structure of the untreated and treated samples was quantitatively analyzed by peak deconvolution of the amide I region (1585–1710 cm^−1^) using the Origin software [58]. The amide I region was fitted into five Gaussian peaks centered at 1610 cm^−1^, 1625 cm^−1^, 1650 cm^−1^, 1670 cm^−1^, and 1690 cm^−1^ with a constant bandwidth (FHWM = 25 cm^−1^) and varied peak intensities to achieve the best fitting (R^2^ > 0.98). The percentage of the secondary structure composition of each fiber type was evaluated by integrating the area of each deconvoluted peak (1650 cm^−1^ for random coils; 1625 cm^−1^ and 1690 cm^−1^ for the β-sheets) normalized to the total area of the amide I region of the fitted curve [59].

### 2.6. Aqueous Stability and Water Update Capability

The aqueous stability of pure SF and SF-ICG fibers was assessed. Three replicates of EVT fiber samples were weighed to obtain their initial dry mass (M_0_), then immersed in 100 μL of DI water at room temperature for 72 h. After the supernatant was removed, the samples were air dried for 24 h at room temperature under the fume hood before their remaining dry mass (M) was recorded. The stability was evaluated by the percentage of mass remaining (%).

(1) $$Mass\;remaining\left(\%\right)=\frac{M-M_{0}}{M_{0}}\times 100\%$$

To measure the water uptake capability, pure SF and SF-ICG (0.1% and 0.4%) fibers of mass 2–3 mg were used in the study. First, the dry weight (M) of three replicates for each sample was measured. Subsequently, they were soaked in 100 μL of DI water at room temperature for 1 h. The excess water on the surface was removed by gently tapping it on a Kimwipe, followed by measuring the wet mass (M_w_) of the sample. The water uptake percentage was calculated:

(2) $$Water\;Uptake\left(\%\right)=\frac{M_{w}-M}{M}\times 100$$

### 2.7. Mechanical Test

Mechanical properties of the EVT fibers, electrospun from a 100 μL protein solution, were examined with stress–strain analysis using an Instron 5542 system (Instron Inc., Norwood, MA, USA) and Blue Hill 2 Software (Instron Inc., Norwood, MA, USA), as reported previously [56,57]. E-spun fibers were collected on precut aluminum holders, followed by ethanol vapor treatment. A load of 50 N was applied, and the load vs. stretching displacement curves were collected at a data acquisition rate of 120 Hz. The ultimate tensile strength, ultimate strain, and Young’s modulus were obtained for the pure SF and SF-ICG fibers. For each fiber type, three replicates were prepared, and the data were reported as a mean value from the measurements.

### 2.8. Differential Scanning Calorimetry (DSC)

The effect of ICG on the decomposition temperature and enthalpy of SF-ICG fibers was analyzed using a DSC 822 calorimeter (Mettler-Toledo, Columbus, OH, USA). Three replicates of the EVT fiber samples of each fiber type, electrospun from a 100 μL protein solution, were examined. The samples were placed in an enclosed aluminum pan, and the measurements were carried out by heating the samples from room temperature to 400 °C under a nitrogen flow rate of 100 mL/min and a constant heating rate of 10 °C/min. The enthalpy of decomposition (J/g) was derived from the integration of the peak around 285 °C using the STARe 9.01 software, following this equation:

ΔH = ∫(dQ/dt) dt,

where ΔH is the enthalpy change of decomposition, and dQ/dt represents the heat flow measured by the DSC as a function of time.

### 2.9. Heat Evolution

An open calorimetry system was developed to measure the heat evolution. Ethanol vapor-treated pure SF, SF-ICG 0.1%, and SF-ICG 0.4% fibers electrospun from a 100 μL protein solution were shaped into loose fiber balls of 2–3 mm in diameter. After the measurement of dry weight, each fiber ball was confined in a well of a 96-well plate and immersed in 100 μL of DI water for 30 min. For each fiber type, six replicates were prepared, and three were each exposed to 30 s or 60 s irradiation using an 808 nm NIR light source of power 500 mW (Beamqus). The temperature change of water in individual wells was measured before and immediately after the irradiation to evaluate heat evolution while avoiding heat loss to the environment. In the control experiment, the temperature change of water alone was measured under the same irradiation conditions. The heat evolution from each sample was evaluated using the following equation:

(3) $$Q=\frac{m_{water}\times c_{water}\times ∆T_{water}}{mass\;of\;fiber\;ball(g)},$$

where Q is the heat evolved per gram of SF-ICG fiber (in J/g), m_water_ is the mass of the DI water (0.1 g), and C_water_ is the specific heat capacity of water (4.186 J/g °C), assuming that the contribution from the fibers was negligible due to the significant difference between the mass of water and the mass of fibers, and ∆T_water_ is the temperature change of the water in °C.

### 2.10. UV-Vis Spectroscopy

The absorption spectrum of the similarly prepared 3D fiber matrices was measured on a 96-well plate while individual fiber balls were immersed in 100 μL of DI water. The spectra were collected in the range of 290 nm to 1000 nm using a Biotek Synergy H1 microplate reader (Agilent Technologies, Santa Clara, CA, USA). The obtained spectra were baseline corrected, and the absorbance was normalized using the dry weight of the sample. The final spectra were plotted as an average of three sets of measurements for each sample type. 

### 2.11. Home-Built System to Simulate Blood Flow for Bleeding Control Experiment

A home-built system was used to simulate blood flow and demonstrate the control of bleeding using the SF-ICG matrix. It consists of a syringe pump (New Era Pump Systems Inc., Farmingdale, NY, USA), a 5 mL syringe (BD Luer Lok), a BD 26 G needle, and a glass pipette. The syringe attached to the 26 G needle was filled with bovine blood, which was then connected to the syringe pump. The SF-ICG 0.4% or pure SF fibrous matrices were inserted in the conical area of the precut glass pipette, which was placed around the needle to form a cylindrical coaxial tube. For this study, a 0.5 mL/h flow rate of blood was infused using the syringe pump. An NIR light source of 808 nm and 500 mW power was used to irradiate the fiber balls. The entire experiment was performed inside a fume hood at room temperature.

### 2.12. Statistical Analysis

Data were presented as the mean ± standard error derived from a set of three replicates in all the experiments. One-way analysis of variance (ANOVA) followed by Tukey’s post hoc test was used to compare the variance of the data groups. A two-way analysis of variance (ANOVA) was performed, followed by the Bonferroni post hoc test. A *p*-value of (*p* ˂ 0.05) was considered to be significant. 

## 3. Results

### 3.1. Electrospun Fibers

**Figure 2:**
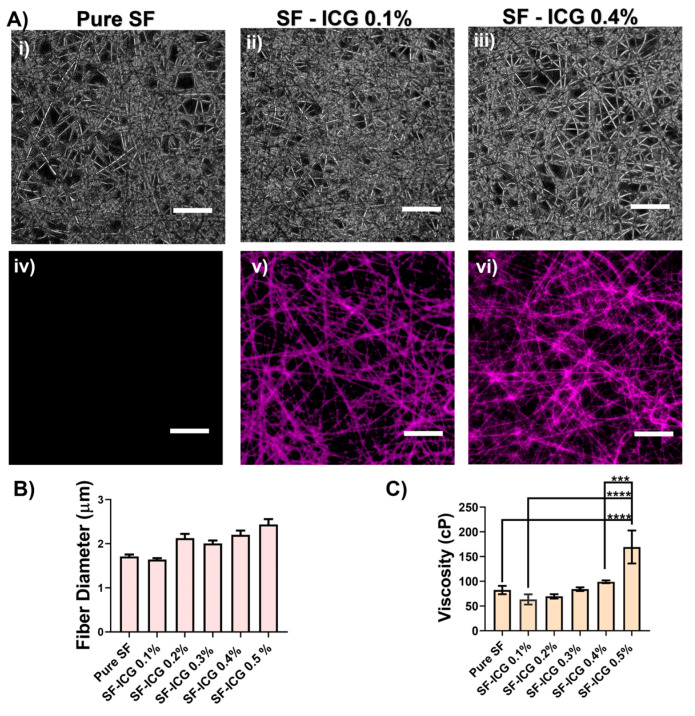
Electrospun SF-ICG fibers at varied ICG weight percentage. (**A**) Phase (i–iii) and fluorescent (iv–vi) images of the fibrous network for pure SF (i,iv), SF-ICG 0.1% (ii,v), and SF-ICG 0.4% (iii,vi). The images were collected at 20x magnification. Scale bar: 10 μm. (**B**) Variation of fiber diameter with ICG percentage. (**C**) Variation of SF solution viscosity with ICG percentage. ***: *p* < 0.001; ****: *p* < 0.0001.

SF and SF-ICG fibers were produced by electrospinning, with a 100 mg/mL SF solution containing ICG at varied weight percentages. As shown in Figure 2A, the fibers formed a dense fibrous network. While no fluorescence was observed in pure SF fibers, increased fluorescence intensity was observed in SF-ICG fibers, with an increase in ICG concentration. The NIR fluorescence arises from ICG, and the uniform and exclusive distribution of fluorescence along the fibers indicates the effective integration of ICG within the SF fibers.

While protein concentration usually impacts fiber diameter [60], our study maintained a constant silk fibroin concentration. The presence of ICG in SF had a moderate effect on fiber diameter. As shown in Figure 2B, adding 0.1% ICG to SF slightly decreased the fiber diameter from 1.71 ± 0.04 μm (pure SF) to 1.64 ± 0.03 μm (SF-ICG 0.1%), and the difference between the two groups was statistically insignificant (*p* = 0.05). A further increase in ICG percentage led to an increase in fiber diameter. SF-ICG 0.4% fibers demonstrated a fiber diameter of 2.20 ± 0.03 μm, which was substantially different (*p* < 0.0001) from both pure SF and SF-ICG 0.1%. The difference in fiber diameter is insignificant among fibers with ICG percentages from 0.2 to 0.6%.

The viscosity of an electrospinning solution is known to affect fiber diameters [55,57,58]. Given the same SF concentration (100 mg/mL), adding 0.1% ICG decreased the solution viscosity to 56.0 cP from 73.1 cP for pure SF (Figure 2C). Although no statistically significant difference was found between the two groups (*p* = 0.88), the reduced viscosity of the SF-ICG 0.1% solution was evident as drops of liquid dripped from the needle’s tip before electrospinning. A small amount of ICG acts as a “contaminant” that disrupts protein–protein interactions, reducing fluid viscosity and may, in turn, reduce the electrospun fiber diameter. At a higher ICG percentage, however, the SF-ICG solution viscosity increased, and the E-spun fiber diameter increased. We surmise that a higher percentage of ICG enabled an adequate number of ICG molecules surrounding and integrating with SF to form a stable structure, which was supported by results from thermal analysis (see below). On the other hand, when the ICG percentage exceeded 0.4%, the SF solution viscosity was too high, causing needle clogging and the formation of multiple jet streams to affect the fiber formation during electrospinning [55,57,58]. Therefore, we chose pure SF, SF-ICG 0.1%, and SF-ICG 0.4% solutions to generate fibers in the following comparative studies.

### 3.2. Effect of Ethanol Vapor Treatment 

**Figure 3:**
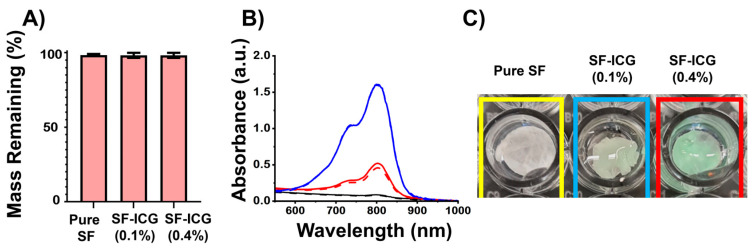
Post-treatment effect on E-spun fibers’ stability. (**A**) Aqueous stability of treated SF and SF-ICG fibers after the fibers were submerged in DI water for 72 h at room temperature. (**B**) ICG stability in pure SF (black), SF-ICG 0.1% (red) and SF-ICG 0.4% (blue) fibers evaluated by UV-Vis absorbance spectra of the fibers before (solid line) and after (dashed line) submersed in DI water for 24 h. (**C**) Photographs illustrating the difference between the pure SF and the SF-ICG fibrous matrices, placed in a 96-well plate in DI water.

As-spun SF and SF-ICG fibers were found unstable in an aqueous environment. After being immersed in deionized (DI) water at room temperature, the fibers dissolved within 20 s. Ethanol vapor treatment (EVT) markedly improved the fibers’ aqueous stability. As shown in Figure 3A, the mass retention of EVT fibers was above 98% for all fiber types even after they were submerged in DI water for 72 h. This is consistent with our previous result [58]. The post-treated fibers also exhibited high stability in the cell culture medium but gradually degraded in the presence of collagenase, making them a promising candidate for use as tissue engineering scaffolds [57,58]. Additionally, there was no visible mass loss when the fibers were immersed in bovine blood.

ICG’s stability in the EVT composite fibers was verified by the absorption spectra of pure SF and SF-ICG fibers (Figure 3B). The characteristic absorption band of ICG in the range of 650–900 nm was observed in the spectra of both SF-ICG 0.1% and SF-ICG 0.4% samples but was absent in the spectrum of pure SF. With the increase in ICG percentage, the intensity of the absorption peak increases. Noticeably, even after 24 h immersion in water, the absorption peak intensity remains unchanged (Figure 3B). A successful and stable integration of ICG molecules within the SF fibers was further verified by the photograph of pure SF, SF-ICG 0.1%, and SF-ICG 0.4% fibrous matrices shown in Figure 3C. 

**Figure 4:**
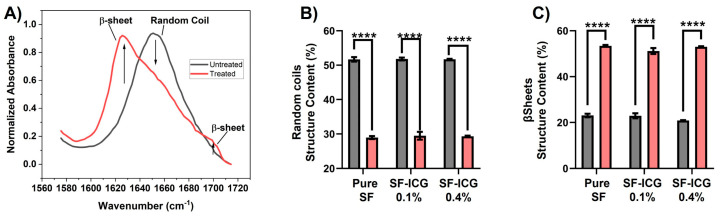
Post-treatment effect on E-spun fibers’ secondary structural transformation. (**A**) The ATR-FTIR spectra of untreated (black) and treated (red) SF-ICG 0.1% fibers in amide I region (1585–1710 cm^−1^). (**B**,**C**) Change of fractional distribution of helix/random coil (**B**) and β-sheet (**C**) in untreated (black) and treated (red) SF and SF-ICG fibers. The difference is statistically significant (**** *p* < 0.0001).

A secondary structural alteration in silk proteins is known to be induced by EVT [58]. The FTIR spectrum in the amide I region (1585–1710 cm^−1^) mostly corresponds to the carbonyl group of the polypeptide backbone and serves as a highly responsive indicator of proteins’ secondary structure [32,61]. The peak centered at 1650 cm^−1^ is representative of helices and random coils, while the peaks at 1620 cm^−1^ and 1690 cm^−1^ are indicative of β-sheets [56,62]. As shown in the spectra of SF-ICG 0.1% fibers in Figure 4A, EVT resulted in a noticeable reduction of the peak at 1650 cm^−1^ and a significant increase of the peaks at 1620 cm^−1^ peak and 1690 cm^−1^, indicating the helix/coil to β-sheet transition. The same spectral changes were observed for pure SF and other SF-ICG 0.4% fibers. The changes were quantitatively analyzed by spectral deconvoluting [58], and the results are summarized in Figure 4B,C. Apparently, the helix/coil structure was predominant in as-spun SF and SF-ICG fibers; the β-sheet component was more than doubled by post-treatment. Therefore, EVT effectively transformed the helix/coil structure to the β-sheets, which contributes to the water stability and the mechanical strength of the post-treated fibers. The secondary structural transition was observed in all fiber types regardless of ICG percentage, consistent with their improved aqueous stability.

### 3.3. Mechanical Properties of SF-ICG Fibers

**Figure 5:**
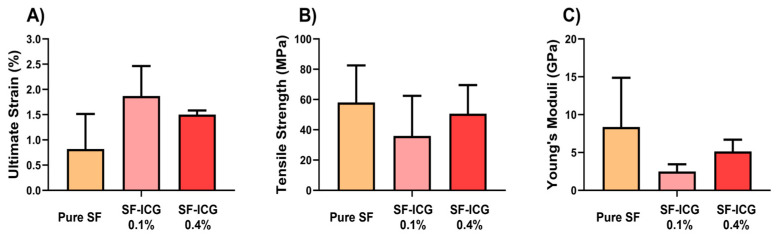
Mechanical properties of the pure SF, SF-ICG 0.1%, and SF-ICG 0.4% fibers. (**A**) Ultimate strain; (**B**) Tensile strength; (**C**) Young’s modulus.

The mechanical characteristics of the EVT fibers were assessed by measuring the stress–strain curves. Figure 5 shows that when 0.1% ICG was added to pure SF, the tensile strength and Young’s modulus dropped, whereas the ultimate strain increased. On the other hand, when ICG was increased to 0.4%, both the tensile strength and Young’s modulus increased at the expense of a decrease in the ultimate strain. Apparently, ICG played distinct roles in SF fiber mechanics when it integrated into SF at a low or high percentage.

We also examined the water uptake capacity (WUC) of the 3D fibrous matrices. The WUC of pure SF, SF-ICG (0.1%), and SF-ICG (0.4%) were 380%, 318%, and 473%, respectively. While the difference in WUC for pure SF and SF-ICG (0.1%) was marginal, the WUC of SF-ICG (0.4%) was significantly higher than that of both pure SF (*p* < 0.025) and SF-ICG 0.1% (*p* < 0.0003), implying that a higher ICG concentration enabled a higher water uptake. The increased aqueous absorptivity of the 3D matrix is favorable for employing it as a wound dressing material.

### 3.4. Thermal Properties of SF-ICG Fibers

**Figure 6:**
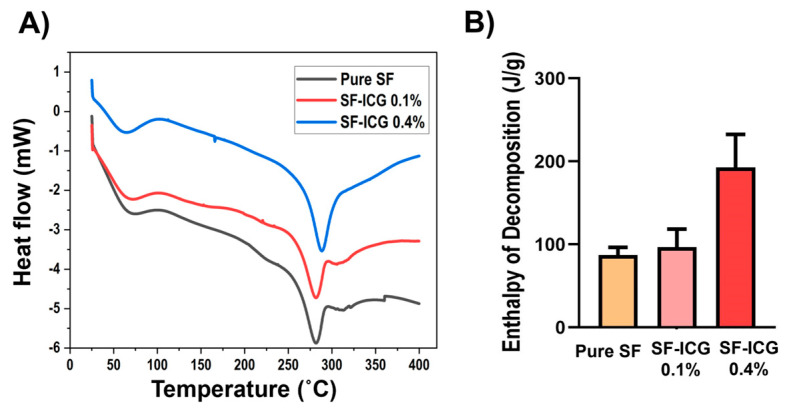
DSC characterization of pure SF, SF-ICG 0.1%, and SF-ICG 0.4% fibers. (**A**) DSC thermograms. (**B**) Enthalpy of decomposition (J/g) derived from integration of the thermal decomposition peak in (**A**).

To examine the impact of ICG on the thermal properties of SF, DSC measurement was carried out. Figure 6A displays the DSC thermograms revealing two endothermic peaks. The first broad peak, centered at 72 °C for SF and SF-ICG 0.1% fibers and 64 °C for SF-ICG 0.4% fibers, is associated with protein denaturation. The second peak, corresponding to the thermal decomposition of the protein fibers, was found at 282 °C for pure SF and SF-ICG 0.1% fibers and 288 °C for SF-ICG 0.4% fibers. The enthalpy of decomposition (J/g) was derived from the integration of the thermal decomposition peak, and the result is shown in Figure 6B. The result suggests that, while a low percentage of ICG did not alter SF fibers’ thermal properties, a high percentage of ICG impinged upon both protein denaturation and protein thermal decomposition. One-way ANOVA analysis showed that the enthalpy of decomposition for SF-ICG 0.4% fibers was significantly different from that of pure SF (*p* < 0.007) and SF-ICG 0.1% (*p* < 0.011). There was no significant difference in the enthalpy of decomposition between pure SF and SF-ICG 0.1%.

### 3.5. Photothermal Response of SF-ICG Fibers

**Figure 7:**
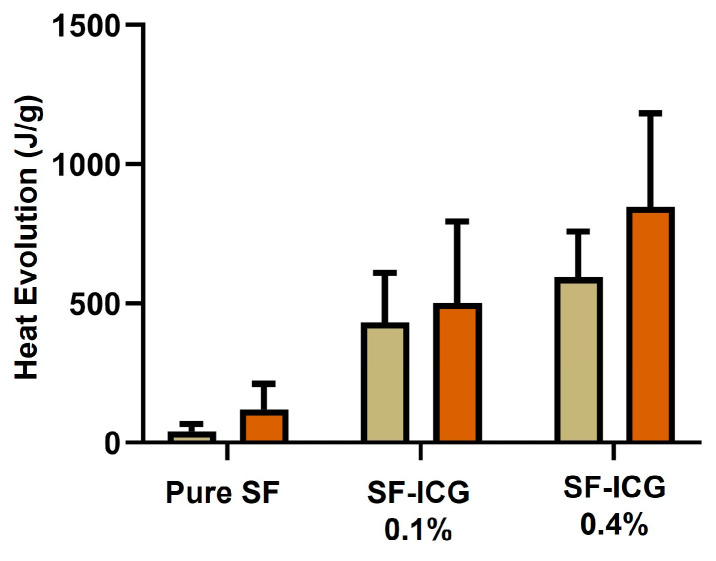
Comparative study of the photothermal response of the pure SF, SF-ICG 0.1%, and SF-ICG 0.4% fibers. Heat evolution was evaluated following NIR irradiation of the fibers for 30 s and 60 s, respectively.

We analyzed the heat evolution of SF and SF-ICG fibers in response to 808 nm NIR irradiation for 30 s and 60 s, respectively. As shown in Figure 7, pure SF fibers produced very little heat even when the light dosage was doubled (30 s vs. 60 s irradiation). For SF-ICG 0.1% fibers, 30 s and 60 s irradiation induced heat evolution of 431.4 J/g and 501.3 J/g, respectively, and the difference between the two groups was statistically insignificant (*p* > 0.05). The heat produced by SF-ICG 0.4% fibers increased significantly, from 593.9 J/g to 846.7 J/g, when the light dosage was increased by increasing the irradiation time from 30 s to 60 s (*p* < 0.05). It is also evident that the increase in ICG percentage in SF fibers notably increased the amount of heat produced. Taken together, the heat produced by SF-ICG fibers increased with ICG percentage; more heat was produced at a higher light dosage. The produced heat is ascribed to ICG’s photothermal property. The stabilized ICG in SF-ICG fibers can absorb NIR light effectively and produce heat when it returns from the excited state to the ground state (see Discussion Section 4).

### 3.6. Application in Bleeding Control 

**Figure 8:**
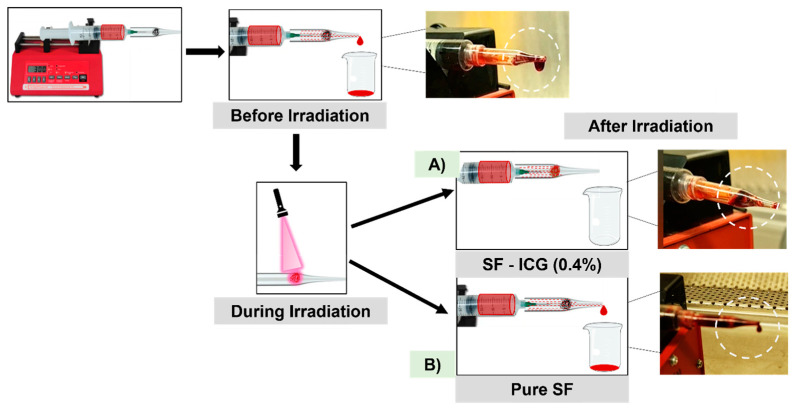
Schematic illustration of the application of (**A**) SF-ICG 0.4% and (**B**) pure SF (control) fibrous matrices in a home-built system mimicking the bleeding of a damaged blood vessel. NIR irradiation activated the SF-ICG 0.4% fibers to effectively stop blood flow (highlighted by the circle in A); pure SF fibers didn’t stop blood flow following NIR irradiation (highlighted by the circle in B). Supplementary Videos are included in Videos S1 and S2.

When water was replaced by bovine blood and uptaken by an SF-ICG 0.4% fibrous matrix, it was observed that 25 s NIR irradiation could induce complete blood coagulation (Figure S1). With the SF-ICG fibers’ stability, durability, and capability of evolving a good amount of heat in response to NIR light, their application toward bleeding control was explored using a home-built system to mimic the bleeding of a damaged blood vessel. As shown in Figure 8, bovine blood was pumped from the syringe into the precut glass by a syringe pump. It was observed that before irradiating the inserted pure SF and SF-ICG 0.4% fibers, the blood was able to pass through the fibers into a receptacle at the end of the pipette tip. After irradiating the SF-ICG 0.4% fibers, however, the blood flow reversed instead of passing through the fibers, as depicted in Figure 8A. This is because the blood absorbed by the fibrous matrix was quickly solidified due to the heat generated by ICG’s photothermal response, effectively halting blood flow. It was verified by the negative control using pure SF fibers, which did not stop blood flow following NIR irradiation (Figure 8B). The Supplementary Videos (Videos S1 and S2) showcased the comparative result of utilizing pure SF and SF-ICG 0.4% fibers in the device and demonstrated the efficacy of SF-ICG fibers in managing bleeding following NIR exposure. It implicated the potential of using the material and method in a variety of biomedical applications, such as hemorrhage control during surgical operations and treatments.

## 4. Discussion

Injury and surgery patients need bleeding control to avoid life-threatening blood loss. Despite medical advances, innovative biomaterials that regulate bleeding swiftly and effectively are in high demand. In this work, we employed the cost-effective electrospinning technique to generate SF-ICG composite fibrous scaffolds, owing to SF’s flexibility, biocompatibility, and biodegradability, and the FDA-approved ICG dye’s photothermal characteristics. The fluorescent images of SF-ICG fibers and their absorption spectra indicated the uniform distribution and integration of ICG within the SF fibers. The reconstituted SF fibers are unstable in aqueous environments, in contrast to native SF’s high aqueous stability. It is ascribed to the predominant helix and random coil secondary structures and short peptides in the reconstituted protein fibers, evidenced by the signature peaks in FTIR spectra (Figure 4). These structures are relatively hydrophilic and water soluble [26,58,63]. The application of EVT induced the helix/coil to β-sheet secondary structural transition in the fibers. This transformation occurs when ethanol acts as a plasticizer to remove intermolecularly bound water molecules, facilitating hydrogen bond formation between peptides that favors their rearrangement into β-sheet crystallites [64]. These crystalline domains are hydrophobic and responsible for the mechanical strength and aqueous stability of SF fibers [25,63,65]. The treatment was effective for both SF and SF-ICG fibers and increased β-sheet conformation 2.3-, 2.2-, and 2.5-fold for pure SF, SF-ICG 0.1%, and SF-ICG 0.4%, respectively. The difference is insignificant. Thus, the presence of ICG did not arrestingly impact the secondary structural transition. Similar EVT-induced secondary structural changes were also observed in SF-CNT fibers in our previous studies. However, the percentage of secondary structural change is different [58]. The discrepancy is attributed to the difference in electrospinning conditions, such as flow rate and fiber type, among others. 

The secondary structural change also resulted in ICG stability in the SF fibers. ICG did not leach out even after the SF-ICG fibers were immersed in water for 24 h (Figure 3B). This is attributed to the strong interaction between SF and ICG at the molecular level. The β-sheet crystalline domains of silk fibroin contain Gly-X repeats, with X being Ala in 65%, Ser in 23%, and Tyr in 9%. These amino acid residues can form hydrogen bonds with the sulfonate group of ICG. Additionally, the high tyrosine content in SF can enhance interactions with the aromatic motif of ICG via p-p stacking in a hydrophobic environment rich in GA repeats, similar to the reported SF interaction with rhodamine [66]. The EVT-induced β-sheet formation may help trap a significant amount of ICG within the spaces. Thus, ICG is an effective additive to enhance the stability of the SF-ICG composite fibers. On the other hand, the ICG molecule is distinctive from SF proteins. As a foreign species, it disrupts SF proteins’ 3D structure and weakens the protein–protein interaction within SF fibers. These opposing roles of ICG account for the distinct properties of SF-ICG at different ICG percentages. In SF-ICG 0.4% fibers, the DSC analysis suggests that a high percentage of ICG relaxes the protein’s three-dimensional structure, evidenced by the decrease in protein denaturation temperature. A higher percentage of ICG also led to an increase in thermal decomposition temperature, ascribing to the strong interaction between SF and ICG to enhance the fiber’s stability against thermal decomposition. At a low percentage, the effect of ICG on SF’s thermal properties is trivial. However, ICG as an impurity disrupted the protein–protein interaction within SF fibers, hence increasing fiber fragility and impairing fiber mechanics.

The networked 3D matrices of SF or SF-ICG fibers exhibited distinct characteristics depending on their moisture content. When dried, the matrix appeared hard and brittle; however, upon wetting, it became soft and durable. This transition indicates the sponge-like nature of the matrix, demonstrating a remarkable capacity for water uptake. The presence of ICG, a lipophilic anion, within the SF fibrous network increased the local ion concentration, drawing water into the 3D matrix via osmosis and thereby enhancing water uptake. The SF-ICG 0.4% fibers were shown to uptake >470% water, higher than the water uptake capacity of pure SF and SF-ICG 0.1% fibrous matrices. This high capacity for water absorption is crucial for the material’s effectiveness in applications such as wound dressing.

The SF-ICG composite fibers were observed to release a considerable amount of heat following a short-term NIR exposure. This is because ICG within the fibers absorbs NIR light, converting it into thermal energy via a non-radiative relaxation process. Upon immersion of the SF-ICG matrix in water and subsequent irradiation, the released energy was transferred to water, increasing water temperature. When bovine blood replaced water, the heat released from the SF-ICG fibers proved adequate to solidify the blood, effectively halting blood flow. While the flow rate in this initial study is lower than the physiological flow rates of blood, the result suggests the promising potential of the light-activatable fibers for use in blood clotting hemorrhage wounds to help stop uncontrollable bleeding. Most recently, we successfully generated silk-ICG films, which also demonstrated comparable blood coagulation effects. These results laid the groundwork to carry out further studies of SF-ICG composites as easy-to-use and light-activated wound dressings and demonstrate their clinical utility in physiologically relevant and in vivo animal studies. 

## 5. Conclusions

In this study, we employed the simple and cost-effective electrospinning technique to generate SF-ICG fibrous matrices with controlled chemical composition. The ethanol vapor post-treatment facilitated β-sheet formation in SF fibers, thereby enhancing the fibers’ aqueous stability and mechanical strength while improving fluid uptake and preventing ICG leaching due to the enhanced SF-ICG interaction. Notably, the ICG-integrated SF fibers generated a significant amount of heat under NIR irradiation, effectively accelerating blood coagulation and flow cessation in an in vitro model that mimicked a damaged blood vessel. Additionally, an SF-ICG 0.4% fibrous matrix demonstrated excellent swelling ability and achieved blood coagulation within 25 s of NIR irradiation, showcasing their potential and efficiency in hemorrhage control. Further exploration of using different blood flow rates and assessment via in vivo animal models are anticipated to provide insights into the clinical utility. The established platform will enable easy incorporation of bioactive and drug molecules into SF-ICG fibers to explore the enhancement of therapeutic effects for wound healing and other medicinal applications.

## Data Availability

The data presented in this study are available on request from the corresponding author.

## Supplementary Materials

The following supporting information can be downloaded at https://www.mdpi.com/article/10.3390/jfb15090272/s1, Figure S1. Bovine blood coagulation on SF and SF-ICG fibrous matrices; Videos S1 and S2: The effect of NIR irradiated pure silk and SF-ICG 0.4% fibrous matrices in halting blood flow in an in vitro model that mimicked a damaged blood vessel.
