# Supplementary material for: Electrospun Silk-ICG Composite Fibers and the Application toward Hemorrhage Control
Source: J Funct Biomater. 2024 Sep 19;15(9):272. doi: 10.3390/jfb15090272 (PMC11433354; doi:10.3390/jfb15090272)
Supplement: Supplementary file 1 [file jfb-15-00272-s001.zip › jfb-3160879-supplementary.pdf]

Supplementary Information

Electrospun Silk-ICG Composite Fibers and the Application toward Hemorrhage Control

*Ayesha Siddiqua[1], Elwin Clutter[1], Olga Garklavs[2], Hemalatha Kanniyappan[1], Rong R. Wang[1]**

[1]Department of Chemistry, Illinois Institute of Technology, Chicago, IL, 60616, US

[2]City Colleges of Chicago - Wilbur Wright College, Chicago, IL, 60616, US

* Corresponding Author: wangr@iit.edu (R. Wang); Tel:1-312-567-3121

S1-* Blood Coagulation on Silk-ICG Fibrous Matrix*:

The pure SF, SF-ICG 0.1%, and SF-ICG 0.4% fibrous matrices were used to carry out the bovine blood coagulation experiments (**Figure S1**). For all samples, the fiber matrices were soaked with a 10 μL drop of blood that was immediately absorbed. It was found that upon the NIR light exposure, the SF-ICG 0.4% matrix was able to solidify the blood within 25 s of irradiation. In comparison to the control (unirradiated), the irradiated SF-ICG 0.4% matrix looks compact, dry, and turned into a darker shade indicating successful coagulation of the blood (**Figure S1A**). On the other hand, for SF-ICG 0.1% fibrous matrix, negligible change was observed after the 25 s irradiation. Only after the matrix was irradiated for 55 s or more, the blood was completely solidified (**Figure S1B**). In the case of pure silk, no change was observed after 25 s irradiation. Even after 55 s irradiation, no change in the texture or appearance of the fibers was observed in comparison to the control sample (**Figure S1C**).

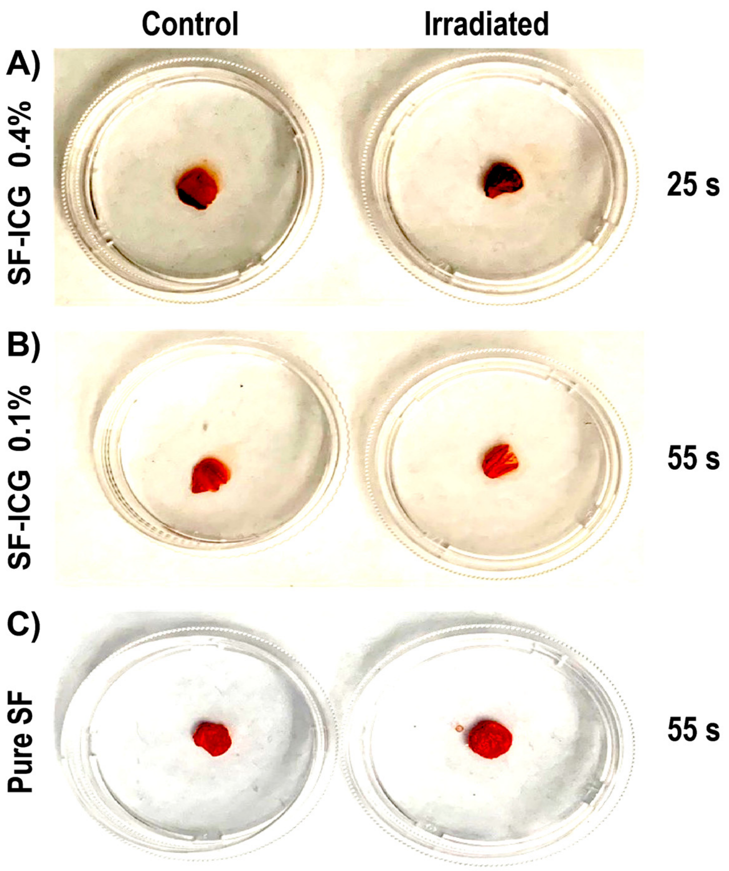

Figure S1: Bovine blood **coagulation** on SF and SF-ICG fibrous matrices. The samples were irradiated using an 808nm NIR laser. A) Blood on SF-ICG 0.4% after 25 s irradiation; B) Blood on SF-ICG 0.1% after 55 s irradiation; and C) Blood on pure Silk after 55 s irradiation. Controls are the same fibrous matrices soaked with blood but without irradiation. The solidification of the blood droplet is demonstrated by the dark red color in the SF-ICG (0.4%) scaffold, compared to the control Pure SF, which remains a normal red color.

***S2.* Supplementary videos:**

Videos to illustrate the effect of pure silk and SF-ICG 0.4% fibrous matrices in halting blood flow in response to NIR irradiation. A home-built system was used (**Fig. 8**) to mimic the bleeding of a damaged blood vessel.

Video S1: for pure SF matrix,
https://drive.google.com/file/d/17A07Txk-Aisco2rJOhpRLsp7NgD7fS9b/view?usp=sharing

Video S2: for SF-ICG 0.4% matrix,
https://drive.google.com/file/d/11CLsBerysUjO1iYwyruPYdQPg9bwevR1/view?usp=sharing
